# Phospholipase D4 as a signature of toll-like receptor 7 or 9 signaling is expressed on blastic T-bet + B cells in systemic lupus erythematosus

**DOI:** 10.1186/s13075-023-03186-5

**Published:** 2023-10-16

**Authors:** Ken Yasaka, Tomohide Yamazaki, Hiroko Sato, Tsuyoshi Shirai, Minkwon Cho, Koji Ishida, Koyu Ito, Tetsuhiro Tanaka, Kouetsu Ogasawara, Hideo Harigae, Tomonori Ishii, Hiroshi Fujii

**Affiliations:** 1 Department of Rheumatology, Tohoku University Hospital, 1-1 Seiryo-Machi, Aoba-Ku, Sendai, Miyagi 980-8574 Japan; 2 Research and Development Department, Ginkgo Biomedical Research Institute, SBI Biotech Co., Ltd., Tokyo, Japan; 3 Department of Immunobiology, Institute of Development Aging and Cancer, Tohoku University, Sendai, Miyagi Japan; 4 Division of Nephrology and Hypertension, Tohoku University Hospital, Sendai, Miyagi Japan; 5 Department of Hematology, Tohoku University Graduate School of Medicine, Sendai, Miyagi Japan

**Keywords:** Phospholipase D4, Systemic lupus erythematosus, Autoreactive B cell, Toll-like receptor, Plasmacytoid dendritic cell

## Abstract

**Background:**

In systemic lupus erythematosus (SLE), autoreactive B cells are thought to develop by-passing immune checkpoints and contribute to its pathogenesis. Toll-like receptor (TLR) 7 and 9 signaling have been implicated in their development and differentiation. Although some B cell subpopulations such as T-bet + double negative 2 (DN2) cells have been identified as autoreactive in the past few years, because the upregulated surface markers of those cells are not exclusive to them, it is still challenging to specifically target autoreactive B cells in SLE patients.

**Methods:**

Our preliminary expression analysis revealed that phospholipase D4 (PLD4) is exclusively expressed in plasmacytoid dendritic cells (pDCs) and B cells in peripheral blood mononuclear cells (PBMCs) samples. Monoclonal antibodies against human PLD4 were generated, and flow cytometry analyses were conducted for PBMCs from 23 healthy donors (HDs) and 40 patients with SLE. In vitro cell culture was also performed to study the conditions that induce PLD4 in B cells from HDs. Finally, recombinant antibodies were synthesized from subpopulations of PLD4 + B cells from a patient with SLE, and their antinuclear activity was measured through enzyme-linked immunosorbent assay.

**Results:**

pDCs from both groups showed comparable frequency of surface PLD4 expression. PLD4 + B cells accounted for only a few percent of HD B cells, whereas they were significantly expanded in patients with SLE (2.1% ± 0.4% vs. 10.8% ± 1.2%, *P* < 0.005). A subpopulation within PLD4 + B cells whose cell size was comparable to CD38 + CD43 + plasmablasts was defined as “PLD4 + blasts,” and their frequencies were significantly correlated with those of plasmablasts (*P* < 0.005). PLD4 + blasts phenotypically overlapped with double negative 2 (DN2) cells, and, in line with this, their frequencies were significantly correlated with several clinical markers of SLE. In vitro assay using healthy PBMCs demonstrated that TLR7 or TLR9 stimulation was sufficient to induce PLD4 on the surface of the B cells. Finally, two out of three recombinant antibodies synthesized from PLD4 + blasts showed antinuclear activity.

**Conclusion:**

PLD4 + B cells, especially “blastic” ones, are likely autoreactive B cells undergoing TLR stimulation. Therefore, PLD4 is a promising target marker in SLE treatment.

**Supplementary Information:**

The online version contains supplementary material available at 10.1186/s13075-023-03186-5.

## Background

Systemic lupus erythematosus (SLE) is an autoimmune chronic disease characterized by the presence of multiple autoantibodies in the circulation [1]. Those autoantibodies are believed to play pathogenic roles by forming immune complexes, thereby depositing on organ tissues [2, 3]. Therefore, autoantibody-secreting cells or their precursors, autoreactive B cells, would be promising treatment targets. Many studies have focused on molecular mechanisms in which autoreactive B cells are generated and mature by-passing tolerogenic checkpoints embedded in the immune system. Toll-like receptor (TLR) 7 and TLR9, pattern-recognition receptors recognizing single-strand RNA and CpG motifs of DNA, respectively, are implicated in the abnormal development of autoreactive B cells in both the bone marrow and the periphery [4, 5]. As many of the autoantibodies detected in SLE are reactive to nucleic acids or proteins associated with them, autoreactive B cells in SLE would probably involve TLR7 or TLR9 during their development and differentiation as suggested in previous studies [6–8].

A canonical study indicated that transgenic RF + autoreactive B cells require TLR9 stimulation for activation [6]. Recent evidence suggests that TLR7 signaling plays a pivotal role in generating pathological autoreactive B cells in both mice and humans [7, 8]. Thus, some markers, as it were, TLR signatures that can distinguish B cells having undergone TLR 7 or TLR9 signaling, would be very useful for detecting and targeting pathological B cells [9].

Recently reported B cell populations called double negative 2 (DN2) cells (IgD-, CD27-, CD11c +  + , and CXCR5^lo^) or CD11c +  + T-bet + B cells, which are expanded in patients with SLE [10, 11] have been proven to encompass autoreactive ones and believed to be generated through TLR7 or TLR9 stimulations with the help of some cytokines [12, 13]. However, the surface markers upregulated in DN2 cells such as CD95, CD11c, or CXCR3 are not unique to the population, which makes it challenging to specifically target them.

Phospholipase D4 (PLD4) is a molecule located on endo-lysosome in B cells and plasmacytoid dendritic cells (pDCs) [14]. A meta-analysis of genome-wide association studies suggested that a *PLD4* single nucleotide polymorphism was one of the risk alleles of SLE [15]. At present, PLD4 is believed to functions as an exonuclease to degrade RNA and DNA, thereby regulating the amounts of ligands for TLR7 and TLR9 [14, 16]. However, whether PLD4 is also expressed on the cell surfaces, whether its expression levels could vary by cell activation status, or how it is related to autoimmune pathogenesis, especially in SLE, have been unknown.

In this study, during detailed expression analysis combined with the previous data, we developed monoclonal antibodies against human PLD4. Using these antibodies, we analyzed the surface expression of PLD4 in peripheral blood mononuclear cells (PBMCs) from healthy donors (HDs) and patients with SLE by flow cytometry. We found that the majority of pDCs and only a few percent of B cells were positive for PLD4 and that any other lymphocytes did not express PLD4 on the surface. In SLE, the frequencies of PLD4 + pDCs were comparable to HDs, whereas PLD4 + B cells were highly expanded. In addition, a subpopulation, which was defined by the cell size, highly overlapped with DN2 cells.

Furthermore, our in vitro study demonstrated that TLR7 or TLR9 stimulation with or without B cell receptor (BCR) engagement could substantially induce PLD4 on B cells from HDs. Therefore, we propose that PLD4 + B cells are possibly TLR-stimulated autoreactive ones and promising treatment targets in SLE.

## Methods

### Experimental model and participant details

A total of 23 adult HDs were recruited for peripheral blood acquisition. Samples from 19 and 9 donors were used for the ex vivo flow cytometric analysis and in vitro stimulation assays, respectively. Forty patients with SLE who met the ACR 1997 criteria with or without active disease states between 2018 and 2021 and who consented to participate in the study were recruited. The culture and repertoire analysis assay recruited one patient with SLE who had a disease flare with active nephritis and elevation of anti-dsDNA antibodies and anti-Sm antibodies.

### Generation of monoclonal antibodies against PLD4

Anti human PLD4 monoclonal antibodies (mAbs), T1S-mAbs, were generated by immunizing female BALB/c mice with purified recombinant fusion proteins in which the extracellular domain of the PLD4 molecule is bound to the Fc region of mouse immunoglobulin (Ig) G2a (the cDNA sequence is described in Additional file 1). The comprehensive methods are described in detail in Patent No. WO2013115410 (https://patentscope.wipo.int/search/ja/detail.jsf?docId=WO2013115410).

### PBMC isolation and naive B cell isolation

Up to 30 mL of peripheral blood was acquired with heparin-coated tubes. PBMCs were isolated by the Ficoll gradient method using Ficoll-Paque™ (Thermo Fisher Scientific, Waltham, MA, USA). Following this, PBMCs were suspended in complete RPMI 1640 medium (Sigma-Aldrich, MO, USA) supplemented with ampicillin/streptomycin and 10% fetal bovine serum (FBS, SERANA, NY, USA), followed by red blood cell (RBC) lysis reaction with a RBC lysis buffer (Sigma-Aldrich). The remaining PBMCs with RBCs removed were used for the flow cytometry, cell culture, or naive B cell isolation. Naive B cells were isolated by Naive B Cell Isolation Kit ii, human (Miltenyi Biotec, Gladbach, Germany) according to the manufacturer’s instruction.

### Real-time quantitative PCR analysis of PLD4 gene expression

For the comparison study of PLD4 gene expression among the immune cells and tissue organs, real-time quantitative PCR (RT-qPCR) was performed using ABI Prism 7000 (Applied Biosystems), as previously described [17]. Briefly, cDNA was synthesized from individual cells using a SYBR green qPCR SuperMix-UDG kit (Invitrogen). For the analysis of immune cells, human PBMCs were isolated from apheresis products of healthy blood donors (Memorial Blood Centers of Minnesota, Minneapolis, MN, USA) [17]. To examine the tissue expression, BD™ MTC multiple tissue cDNA panel (Takara Bio) was used. PCR was conducted using ABI Prism 7000 (Applied Biosystems) with the following parameters: 50 °C for 2 min, 95 °C for 10 min, 50 cycles at 95 °C for 15 s, and 60 °C for 1 min. Each transcript was normalized to human glyceraldehyde-3-phosphatedyhydrogenase (GAPDH) gene values to account for sample variation. The primer sequences for the PCR reaction are the following. PLD4 sense primer sequence 1: 5’-AGGACATCCACCGACCTG-3’, PLD4 sense primer sequence 2: 5’-GTGAAAGTCTTCATCGTGCCG-3’, PLD4 anti-sense primer sequence 1: 5’-GCAAAACACCCCTGGTGA-3’, PLD4 anti-sense primer sequence 2: 5’-GTGCTGCTGAAGTAATCCTCC-3’, GAPDH sense primer sequence: 5’-AGCCACATCGCTCAGACAG-3’, GAPDH anti-sense primer sequence: 5’-GCCCAATACGACCAAATCC-3’, β2MG sense primer sequence: 5’-CCACTGAAAAAGATGAGTATGCCT-3’, β2MG anti-sense primer sequence: 5’-CCAATCCAAATGCGGCATCTTCA-3’.

RT-qPCR was also performed to estimate the PLD4 expression in cultured naive B cells. The total RNA was extracted from cultured naive B cells using RNeasy^®^ Plus Micro Kit (Qiagen), and cDNA was then synthesized using ReverTra Ace^®^ qPCR RT kit (Toyobo). cDNA was stored at − 20 °C in TE buffer (pH 8.0) (Nacalai Tesque Inc.). The relative expression of *PLD4* was analyzed by RT-qPCR using SYBR^®^ Green PCR kit (Qiagen). PCR as conducted using a CFX Connect™ Real Time System (BioRad) under the following protocol: incubation at 50 °C for 2 min, 95 °C for 15 min, 40 cycles at 94 °C for 10 s, 55 °C for 1 min, and 72 °C for 1 min. In each sample, cDNA copy numbers were determined by comparing threshold cycles (Ct) with that generated by standard plasmids (copy numbers 10^6^, 10^5^, 10^4^, 10^3^, 10^2^, and 10). RNA expression was normalized by housekeeping genes β2MG.

### Flow cytometry analysis and cell sorting

Isolated 1 × 10^6^ PBMC per tube suspended in staining buffer (PBS containing 1% bovine serum albumin and 10% normal mouse serum (Abcam, Cambridge, UK)) were stained with the following fluorescent antibodies:

- Set 1: CD43 (FITC), CD19 (PerCP-Cy5.5), CD38 (APC), IgD (PE-Cy7), CD24(APC-Cy7), CD3CD14CD16 (V450), CD27 (V500).
- Set 2: CD11c (FITC), CD19 (PerCP-Cy5.5), CD38 (APC) / CD21 (APC), IgD (PE-Cy7), CXCR5 (APC-Cy7), CD3CD14CD16 (V450), CD27 (V500).
- Set 3: CD11c (FITC), BDCA2 (APC), CD14 (APC-Cy7), CD3CD14 (V450), CD16 (V500).
- Set 4: CD4 (FITC), CD8 (PerCP-Cy5.5), CD3 (APC-Cy7), CD14CD19CD16 (V450).

After the incubation on ice for 20 min, the cells in each tube were washed with 1 mL of FACS buffer, suspended by FACS buffer, and split into two tubes for isotype and phospholipase D4 (PLD4) staining. Biotinylated mouse IgG2b (BioLegend, CA, USA) or biotinylated T1S-mAbs were added at 5 μg/mL. After 20 min of incubation on ice, both tubes were washed, suspended again, and then added with PE–streptavidin (BD Pharmingen, CA, USA) at 1 μg/mL. After 15 min of incubation on ice, stained PBMCs were analyzed with a FACS Canto II (BD, Biosciences, NJ, USA).

For in vitro cultured PBMCs, CD20 (PE-Cy7) and CD3CD14CD16 (V450) were used to define B cells. Thereafter, PLD4 was stained as described above. To eliminate dead cells, 5 μL of 7-AAD (BD Pharmingen) was added to 100 μL of the cell solution 5 min before the cytometry analysis. Compensation was done using an antimouse Ig, κ/Negative Control Compensation Particle Set (BD, Biosciences).

For intracellular T-bet staining, 2 × 10^6^ PBMCs were fixed and permeabilized using a True-Nuclear™ Transcription Factor Buffer Set (BioLegend) according to the manufacturer’s instruction. Mouse IgG (Peprotech, NJ, USA) was added to block nonspecific binding at a concentration of 100 μg/mL; then, the fluorescent antibodies against T-bet (APC) or mouse IgG1 (APC) were added. Flow cytometry data were analyzed using the FlowJo software (Treestar).

For the cell culture assay described below, PLD4 + blasts or CD19 + B cells were sorted using a FACS Aria II (BD, Biosciences). Since the number of sortable PLD4 + blasts was not large enough (up to 3,000 cells from 20 mL of peripheral blood), CD19 − and CD3 + , CD14 + , or CD16 + cells (non-B cell PBMCs) were sorted simultaneously as scaffold cells.

### Cell stimulation and culture in vitro

For the PLD4 induction assay, 5 × 10^5^ PBMCs or 1 × 10^5^ naive B cells suspended in complete RPMI1640 medium (10% FBS) were seeded in 96-well non tissue culture-treated plates (Corning, NY, USA). As stimuli, RPMI1640 (no stimulation), 0.15 μM CpG ODN 2006 (Invivogen), 1 μg/mL R848 (Sigma-Aldrich), or 1, 5, or 25 μg/mL AffiniPure F(ab’)2 Fragment Goat Antihuman IgG and IgM (H + L) (Jackson ImmunoResearch, PA, USA) were added, and cells were cultured for 2 days, followed by flow cytometry analysis, as described above.

For the differentiation of B cells into IgG antibody-secreting cells (IgG-ASCs), sorted 1,000 PLD4 + blasts or CD19 + B cells with plasmablasts gated out were seeded together with 10,000 non-B cell PBMCs in the plates. Then, 1 μg/mL R848 or 0.15 μM CpG ODN 2006 was added. After 4 days of culture, IgG-ASCs were counted by ELISpot.

### ELISpot for IgG-secreting cells

IgG-secreting cells were detected by an ELISpot Human IgG (HRP) Kit (Mabtech) according to the manufacturer’s instruction. Briefly, a Multiscreen IP HTS 0.45 μm (Merck Millipore) was coated with 15 μg/mL anti-IgG antibodies overnight at 4 °C. The wells were washed with PBS twice, followed by 1-h blocking by PBS, and 1% BSA. After removing the blocking solution, cultured cells were added to the wells and then incubated at 37 °C under 5% CO_2_ for 16–20 h.

The wells were washed by PBS containing 0.1% Tween 20 (Sigma-Aldrich) (PBS-T) and then the secondary biotinylated anti-IgG antibodies equipped in the kit, which were filtered through a Millex-HV 0.45 μm (Merck Millipore) were added with the concentration of 1 μg/mL. After 2-h incubation, the wells were washed with PBS-T four times, 100 μL of streptavidin–HRP was added, and the plate was incubated for 1 h. After washing the plate, 100 μL of the substrate for ELISpot (Mabtech) was added. The spots were developed for 15–20 min, and the development was stopped by washing the wells extensively in tap water. The plates were sent to MINERVATECH for spot counting.

### BCR repertoire analysis of cultured B cells

BCR repertoire analysis was performed as previously described for T cell repertoires [18]. Briefly, cultured B cells were harvested, and washed with PBS by centrifugation. RNA was extracted using an RNeasy Mini spin column (Qiagen), and cDNA libraries were then made by the following two-step method: first-strand cDNA was synthesized with oligo(dT) primers and SuperscriptTM III Reverse Transcriptase (Invitrogen), and second-strand cDNA was then synthesized with RNase H, T4 DNA polymerase (Invitrogen).

Adapters, in the short strand of which Ts were replaced by Us, were bound to the cDNA libraries. Uracil DNA Glycosylase (Takara) was added to digest the short strand and generate single-chain adapter ligations. The libraries added with the adapters added were amplified by PCR. A next-generation sequencer (Illumina) was used in the amplified products (BCR libraries) according to the manufacturer’s instruction. The result of sequencing was analyzed using IMGT-V-QUEST (https://www.imgt.org/IMGT_vquest/input). Sequences sharing the same V- and J- gene segments with identical CDR3 sequences were identified as identical ones.

### Recombinant monoclonal antibody synthesis

Vector cloning, transfection, and purification of antibodies were performed. The sequences of V(D)J and the constant region (IgG1) were amplified separately by PCR, and the sequence overlapping the vector cloning site was added. The PCR products were individually inserted into the pHEK Ultra expression vector (Takara Bio) using NEBuilder^®^ HiFi DNA Assembly cloning kit (New England BioLabs). The closed vector including the V(D)J + constant region sequence was transformed into One Shot Top10 chemically competent Escherichia coli cells (Invitrogen), followed by culture overnight and plasmid purification with Plasmid Midi kit (Qiagen). The plasmid was transfected into Expi293F™ cells using ScreenFect^TM^UP-293 kit (FUJIFILM Wako). Expi293 cells were proliferated up to 7.5 × 10^7^ cells. After 5 days of culture, the secreted mAbs were purified using Protein G Sepharose 4 Fast Flow Lab Packs (Cytiva). Specifically, the supernatant from the culture cells was added to Pierce™ Disposable 5 mL Polypropylene Column (Thermo Fisher Scientific) loaded with 500 μL of protein G sepharose. After washing the column with 20 mM sodium phosphate, the antibodies were eluted with 100 mM Tris–HCl (pH 9.0).

For the control antibodies for enzyme-linked immunosorbent assay (ELISA), one IgG sequence of B cells isolated from a normal donor was randomly chosen to be subcloned, followed by the synthesis and purification of the mAbs.

### ELISA for antinuclear antibody activity measurement

To test the autoreactivity of the recombinant mAbs, Human antinuclear antibody (ANA) ELISA Kit (CUSABIO) was used following the manufacturer’s instruction. SLE sera which is known to be ANA + and healthy sera were used for control with 101-fold dilution. The mAbs from PLD4 + blasts or mAbs from a control sample were added to the healthy serum at 50 μg/mL. OD450 was measured for each sample.

### Statistical analysis

The nonparametric Mann–Whitney test was used to compare values between groups. Spearman’s rank correlation coefficient was calculated to analyze the correlation between cell frequencies and clinical measurements. Fisher’s exact test was used to analyze the significance of the relative risk of certain clinical manifestations between groups. For some values measured in the same patients or samples at different time points or with different conditions, the Wilcoxon signed-rank sum test was performed. All the analyses were performed using R version 4. 1. 2 (The R Foundation for Statistical Computing, Vienna, Austria). *P* values are indicated as *P < 0.05, **P < 0.01, and ***P < 0.005.

## Results

### *PLD4* is dominantly expressed in pDCs followed by B cells

**Fig. 1:**
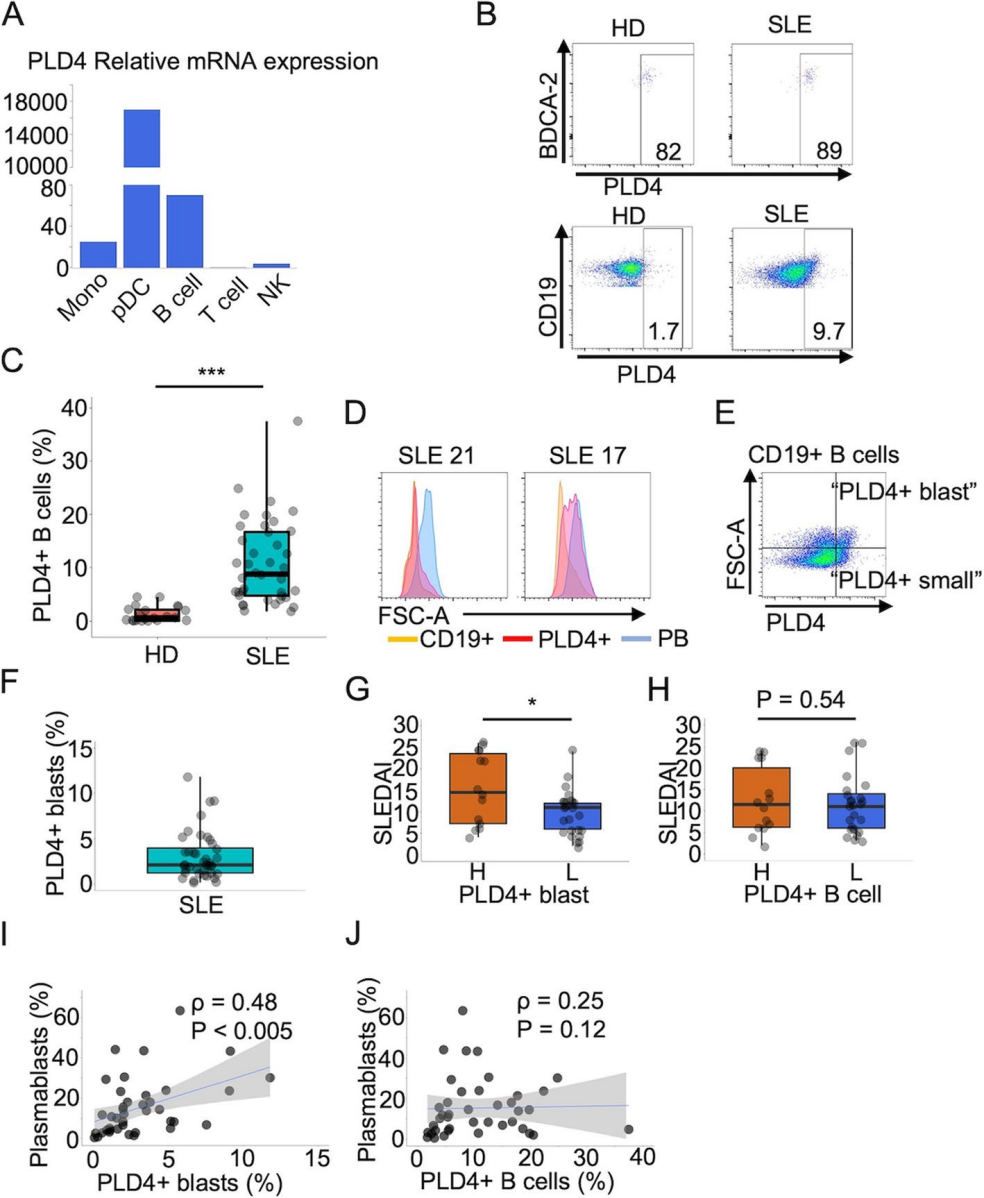
PLD4 expression in immune cells in HDs and patients with SLE. **A** RT-qPCR demonstrated *PLD4* expression among immune cells (*n* = 1). **B** Representative flow cytometry plots showing PLD4 + cells in pDCs and B cells from an HD and a lupus patient. **C** Boxplots showing the ratios of PLD4 + B cells in HDs (*n* = 19) and lupus patients (*n* = 40). **D** FSC-A was analyzed among the populations denoted below the plots. **E** CD19 + B cells were compartmentalized based on the PLD4 expression and FSC-A. **F** A boxplot showing the ratios of PLD4 + blasts in the patients. **G**, **H** The patients were divided by the ratios of PLD4 + blasts (**G**) or PLD4 + B cell (**H**). SLEDAI scores were compared between the two groups. **I**, **J** In lupus patients, the correlation between the ratios of plasmablasts and PLD4 + blasts (**I**) and PLD4 + B cells (**J**) were assessed. *P < 0.05, **P < 0.01, ***P < 0.005, by the Mann–Whitney U test in C, G, and H. In I and J, Spearman rank correlation coefficient (ρ) and *P* values are shown, and the gray shade areas represent the 95% confidence interval of the regression line. Mono, monocytes; NK, natural killer cells

In the previously performed SAGE in which gene transcriptions were compared between human pDCs and monocytes [17], we detected that the *PLD4* gene was dominantly transcribed in pDCs. Subsequently, we tested the mRNA expression of *PLD4* in peripheral immune cells by performing a RT-qPCR on pDCs, B cells, T cells, monocytes, and natural killer cells. The expression of *PLD4* was the highest in pDCs followed by in B cells with a far lesser degree (Fig. 1A). Then, we examined *PLD4* expression by organs. Using cDNA panels from multiple tissues, a RT-qPCR was conducted, in which spleen and peripheral white blood cells exhibited the highest mRNA expression of *PLD4* (see supplementary Fig. 1A (Additional file 2)). Taken together, *PLD4* is highly expressed in pDCs and to a lesser extent B cells, and its expressions are concentrated on immune-related organs.

### PLD4 + B cells are significantly expanded in SLE

Then, we investigated the cell surface expression of PLD4 in PBMCs. We developed monoclonal antibodies against PLD4 (T1S-mAb). Using them, we first performed flow cytometry on PBMCs from HDs (see supplementary Fig. 1B (Additional file 2)). While the majority of pDCs were positive for PLD4, PLD4 + B cells accounted for only a few percent of CD19 + B cells (Fig. 1B). We could not detect any surface PLD4 expression in other immune cells (*n* = 3, see supplementary Fig. 1C (Additional file 2)), which was consistent with mRNA expression analysis. Subsequently, we tested PBMCs from SLE. We recruited 40 patients with SLE, and most (32, 80%) had newly onset and untreated SLE (Table 1). While the frequencies of PLD4 + pDCs were comparable to HDs, PLD4 + B cells were significantly expanded in SLE (Fig. 1C). B cells in SLE have various characteristics. For instance, transitional B cells, double negative (DN) cells, and plasmablasts are increased in patients with SLE compared with healthy controls [19–21]. Consistent with previous reports, DN cells and plasmablasts were significantly more abundant in our cohort than in HDs. In addition, although it was not statistically significant, transitional B cells were more frequent in SLE, with some patients showing a drastic increase of up to 30% (see Supplementary Fig. 2A, B (Additional file 3)). When we looked at cell compositions of the expanded PLD4 + B cells in patients with SLE, transitional B cells, memory B cells, and DN cells accounted for larger percentages than they did in the whole B cells. However, PLD4 + B cells barely contained plasmablasts (see supplementary Fig. 2C (Additional file 3)). Collectively, PLD4 + B cells are expanded in patients with SLE, and they are unique in terms of cell composition.

**Table 1:** Patient demographics

	HD	SLE
n	19	40
Female	11 (58%)	37 (93%)
Age^a^	31 (20–59)	42 (22–76)
SLEDAI^b^		12(2–49)
Treatment naive		32 (80%)
Treatment		
PSL		7 (18%)
HCQ		2 (5%)
TAC		3 (7.5%)
AZA		1 (2.5%)

*PSL* Predonisone, *HCQ* Hydroxychloroquine, *TAC* Tacrolimus, *AZA* Azathioprine

^a^Mean and range

^b^Mean and range

### “PLD4 + blasts” phenotypically and functionally overlap with DN2 cells

To decipher the uniqueness of PLD4 + B cells, we focused on their cell size represented by FSC-A presuming that it roughly reflects the activation status of cells [19]. Of the entire B cell population, plasmablasts showed the largest cell sizes. In some patients with SLE, fractions of PLD4 + B cells were comparable to plasmablasts in terms of cell size (Fig. 1D). As mentioned above, since PLD4 + B cells hardly encompass plasmablasts, we focused on these PLD4 + B cells as large as plasmablasts, thinking that they were more activated than the others. Accordingly, we set a threshold of the 25th percentile of FSC-A of plasmablasts to define such a larger part of PLD4 + B cells and designated them as “PLD4 + blast” and the rest as “PLD4 + small” (Fig. 1E). The ratios of PLD4 + blasts ranged from almost 0 to up to > 10% in patients with SLE (mean = 3.1%, SD = 2.6) (Fig. 1F). When the patients were divided into two groups by the ratio of PLD4 + blasts (higher or lower than mean value [3.1%]), the high group (H) showed significantly higher SLEDAI scores than the low group (L); however, this difference disappeared when divided by the ratio of PLD4 + B cell (blast + small) (Fig. 1G, H). Furthermore, the ratios of plasmablasts and PLD4 + blasts, but not PLD4 + B cells were significantly correlated (Fig. 1I, J) suggesting that PLD4 + blasts should be seen as a distinct population within PLD4 + B cells, which reflects the disease activity of SLE.

**Fig. 2:**
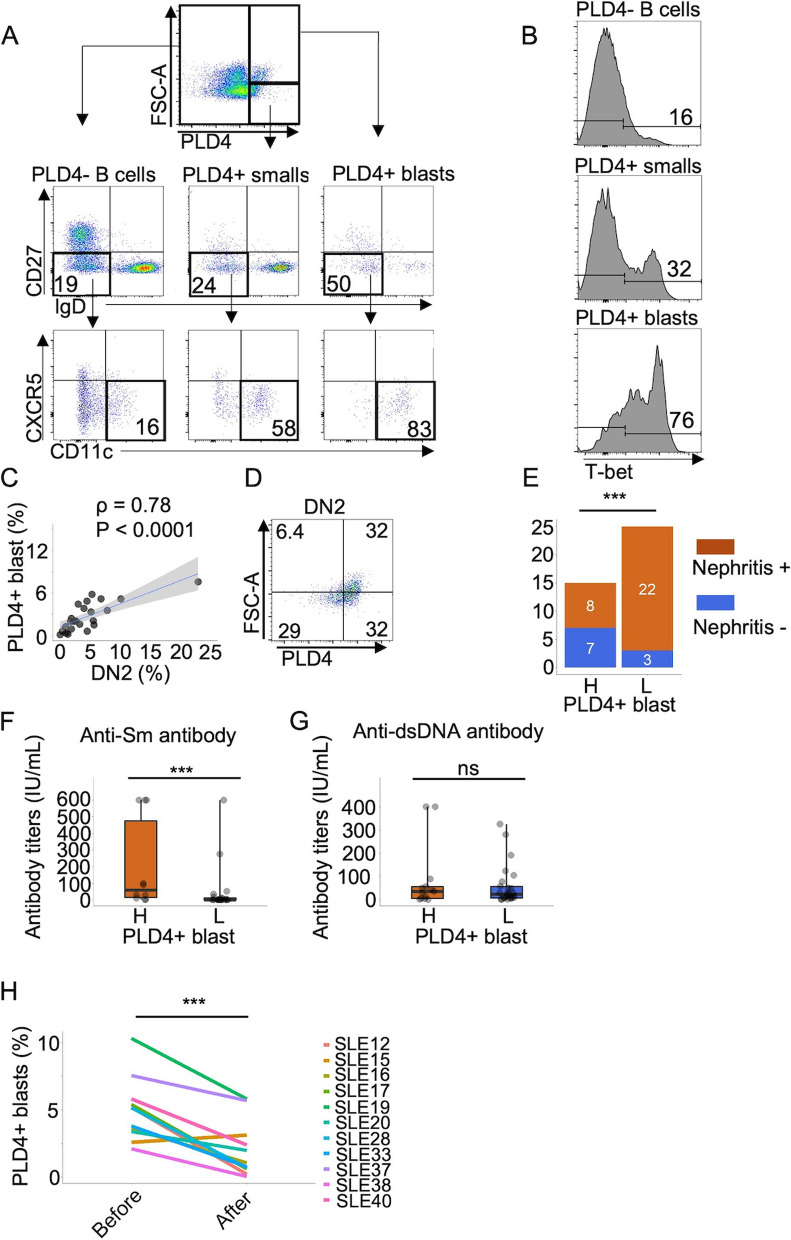
Overlapping between PLD4 + blasts and DN2 cells. **A** Flow cytometry plots assessing the ratios of DN2 cells in each denoted population in a representative patient with SLE. **B** Histograms showing the positivity of intracellular T-bet in each denoted population in a representative patient with SLE. **C** The correlation between the ratios of PLD4 + blast and DN2 cells was assessed. **D** Representative plot in which DN2 cells were assessed by PLD4 and FSC-A. **E**–**G** The patients with SLE were divided as were in Fig. 1G, and the ratios of those with nephritis (**E**) and the titers of anti-Sm antibodies (**F**) or anti-dsDNA antibodies (**G**) were compared between the two groups. ***P < 0.005, ns, not significant. **H** In 11 patients with newly-onset SLE, the ratios of PLD4 + blasts were analyzed before and after immunosuppressive therapies. *P < 0.05, **P < 0.01, ***P < 0.005, by Fisher’s exact test and Mann–Whitney U test in E, F and G, and by Wilcoxon’s signed-rank test in H. In C, Spearman rank correlation coefficient (ρ) and *P* values are shown, and the gray shade areas represent the 95% confidence interval of the regression line. ns, not significant

We further tried to characterize PLD4 + blasts and found that they phenotypically overlapped with DN2 cells, which were featured by IgD-, CD27-, CD11c +  + , and CXCR5 − (Fig. 2A). In addition, PLD4 + blasts, which were not subsumed in the DN population (IgD + or CD27 + ones), were also mostly CD11c +  + and CXCR5- cells suggesting that PLD4 + blasts are highly homogenous in terms of cell ontology (see Supplementary Fig. 3A (Additional file 4)). Consistent with this, intracellular staining of transcription factor T-bet revealed that most of the PLD4 + blasts were T-bet +  + , which is one of the critical features of DN2 cells (Fig. 2B). As expected, the frequencies of PLD4 + blasts and DN2 cells were significantly correlated (Fig. 2C). In addition, within the DN2 cell population, PLD4 + DN2 cells showed larger cell sizes than PLD4- ones (Fig. 2D). As regards clinical manifestations, the high PLD4 + blast group showed higher rates of complication of nephritis and higher titer of anti-Sm antibodies, but not anti-dsDNA antibodies than the low group (Fig. 2E–G). In addition, the ratio of PLD4 + blasts significantly and inversely correlated with serum C3 and C4 levels (see Supplementary Fig. 3B (Additional file 4)). Finally, a significant decrease in PLD4 + blasts was observed after immunosuppressive therapies in some cases (*n* = 11, Fig. 2H). These results suggest that the expansion of PLD4 + blasts reflects certain disease activity as the DN2 cell expansion does.

### TLR7 or TLR9 stimulation is sufficient to induce PLD4 + B cells in HDs

**Fig. 3:**
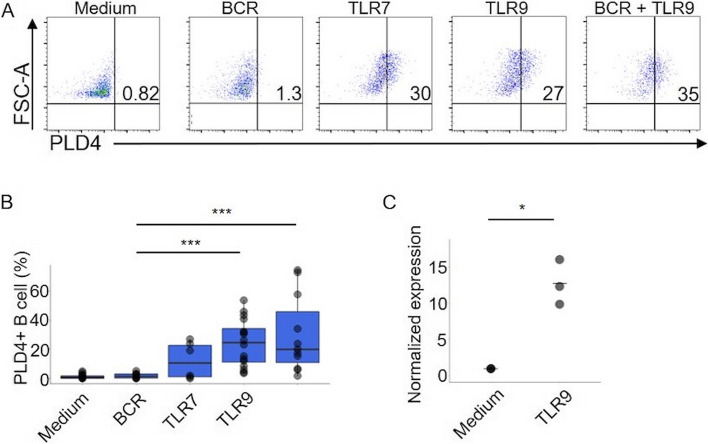
TLR7 or TLR9 stimulation is sufficient to induce PLD4 + B cells in healthy donors. **A**, **B** PBMCs from HDs were cultured in vitro with stimulants. A shows representative plots in one donor. In B, the ratios of induced PLD4 + B cells were represented by dots in the boxplots and compared between the stimulants. **C** Naive B cells isolated from three HDs were cultured with or without TLR9 ligand for 2 days, after which *PLD4* mRNA expressions were measured by RT-qPCR. *P < 0.05, ***P < 0.01, ***P < 0.005, by Mann–Whitney U test in B and by the Wilcoxon signed-rank test in C

Then, we next asked whether the surface PLD4 expression levels in B cells vary according to activation states. Isolated PBMCs from HDs were stimulated by anti-IgG/IgM (BCR cross-linking), TLR7 or TLR9 ligands alone, or BCR + TLR ligands followed by flow cytometry analysis on day 2. Strikingly, PLD4 + B cells were not induced by BCR signaling alone, whereas TLR alone or BCR + TLR stimulations significantly induced PLD4 + B cells (Fig. 3A, B). When naive B cells were isolated, and then stimulated by TLR9 ligand, mRNA levels of *PLD4* significantly increased compared with the unstimulated (Fig. 3C). Therefore, the elevation of *PLD4* expression after TLR7 or TLR9 stimulation was not caused by external factors such as cytokines from stimulated pDCs but by B cell-intrinsic de novo synthesis in response to TLR stimulation. In most of the samples, the combination of BCR + TLR9 stimulation could induce PLD4 + B cells more than TLR9 alone, suggesting the synergized effect of the downstream signaling pathway (Fig. 3B).

### Synthesized IgG antibodies from the largest repertoires of PLD4 + blasts showed antinuclear activity

**Fig. 4:**
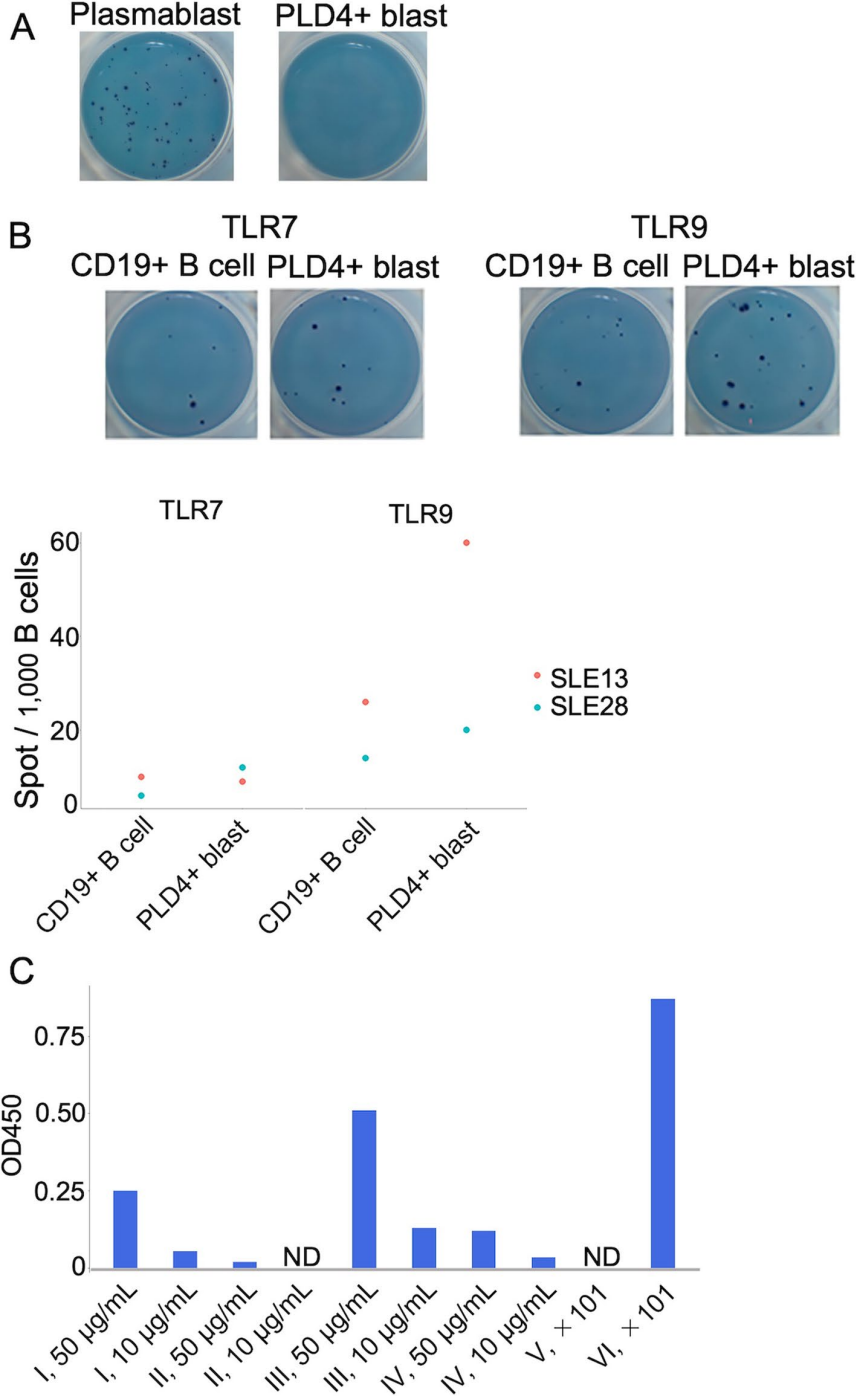
PLD4 + blasts can differentiate into antibody-secreting cells and produce autoantibodies. **A** Plasmablasts and PLD4 + blasts were sorted and directly seeded onto PVDF membrane, followed by IgG ELISpot. **B** CD19 + B cells and PLD4 + blasts were sorted from two patients with SLE and cultured in vitro with TLR7 or TLR9 ligands for 4 days. The representative ELISpot results are shown in the pictures in the upper row and the measured number of spots are shown in the graph in the lower row. **C** The bar graphs show OD450 readings generated by antinuclear antibody enzyme-linked immunosorbent assay (ELISA). Each sample was tested in duplicate. I = VH1-18–01 J + KV 1–16-02, II = VH4-34–01 + KV 2–30-02, and III = VH1-18–01 + KV 1–5-03. IV represents randomly amplified monoclonal antibody from one healthy donor. V, serum from a healthy donor. VI, serum from a patient with SLE. ND, not detected

Given their correlation with certain disease states and overlap with DN2 cells, PLD4 + blasts were speculated to be likely autoreactive and involved in the autoantibody production in SLE. First, ELISpot was performed to assess PLD4 + blasts from two SLE patients for the capacity to produce IgG antibodies. While PLD4 + blasts showed no capacity to produce IgG (Fig. 4A), when cultured with TLR7 or TLR9 ligands, they exhibited more vigorous production of IgG antibodies than the whole CD19 + B cells (Fig. 4B, C).

Subsequently, we tried to test the autoreactivity of PLD4 + blasts. Culturing the cells and measuring the produced antibodies are frequently employed to examine the autoreactivity of B cell populations of interest. However, this method is challenging in terms of reproducibility and sensitivity, particularly with a small number of cells. To avoid these problems, a BCR repertoire analysis of cultured PLD4 + blasts was performed. PLD4 + blasts were sorted from one patient having SLE flare with increased titers of anti-dsDNA (> 400 IU/mL), anti-Sm (46 IU/mL), and anti-SS-A (> 1200 IU/mL) antibodies and class IV lupus nephritis. PLD4 + blasts accounted for 8.5% in CD19 + B cells. We stimulated the sorted PLD4 + blasts for 4 days with the TLR7 ligand and inflammatory cytokines, promoting B cell differentiation into ASCs. The repertoires of TLR-stimulated PLD4 + blasts were skewed; as a result, the frequencies of the top three repertoires accounted for > 50% of the whole, which is probably due to stimulation and culture and were not included in similarly cultured CD19 + B cell repertoires (see Supplementary tables 2–5 (Additional files 5, 6, 7 and 8)). By reasoning that these amplified repertoires probably accounted for those of secreted antibodies in vitro, recombinant monoclonal antibodies were synthesized from the sequence of the three largest repertoires. Among the three synthesized antibodies, two showed antinuclear activity demonstrated by ELISA (Fig. 4D). Thus, PLD4 + blasts highly likely contain autoreactive cells.

## Discussion

Since many of the autoantibodies detected in SLE react to nucleic acids and proteins bound to them, antigen recognition through BCR and TLR7/9 is believed to be critical for the development of autoreactive B cells. In this study, PLD4 + B cells can be induced simply by TLR7 or TLR9 stimulation but not by BCR engagement alone, suggesting that PLD4 is a “TLR7/9 signature” in B cells. As an exonuclease, PLD4 degrades nucleic acids and reduces the amounts of ligands for TLR7 and TLR9 [16]. Therefore, the induction of PLD4 in our assay may be interpreted as a negative feedback process after TLR stimulation, although we did not assess the signaling molecules responsible for this phenomenon. Although further studies are required to investigate how increased PLD4 expression is involved in SLE pathogenesis, cell surface PLD4 might just reflect the increased synthesis inside the cells, considering that PLD4 has been reported to function at a lower pH as in lysosomes.

To our knowledge, this is the first study to report that PLD4 + B cells are expanded in patients with SLE. Interestingly, the cell compositions of PLD4 + B cells were distinct from those of the entire peripheral B cells, with transitional B, memory B, and DN cells more abundant, whereas plasmablasts were hardly included even in patients with active SLE. The origins and development pathway of PLD4 + B cells should be the next task to address.

While PLD4 + B cells were expanded in nearly all our patients with SLE irrespective of disease states, further analysis allowed us to define “PLD4 + blast” as a unique subset within PLD4 + B cells, which in contrast were expanded in parallel with disease activity and the expansion of plasmablasts and highly overlapped with DN2 cells. Since most of our patients had untreated SLE, we believe that the detected correlations between disease phenotypes and PLD4 + blasts are highly relevant, which was also implicated by decreased levels of PLD4 + blasts after treatment. According to recent theories, it is not only chronic antigenic stimulation but also bystander activation that drives the differentiation of autoreactive B cells into ASCs [22–26]. Thus, PLD4 + blasts, together with plasmablasts, would likely emerge in proinflammatory environments enriched with TLR stimulations or certain critical cytokines such as interleukin-21 or B cell activating factor of the TNF-family (BAFF) as proposed in DN2 cell studies.

Since our BCR repertoire analysis was applied to cultured PLD4 + blasts, the amplified clones did not necessarily reflect the actual repertoire landscape of B cells in SLE. Nevertheless, PLD4 + blasts encompassed antinuclear repertoires, consistent with overlapping with DN2 cells. Moreover, when the PLD4 expression in DN2 cells was analyzed, PLD4 + DN2 cells showed larger cell sizes than PLD4- DN2 cells. Although the cell enlargement is not necessarily attributed to one reason, PLD4 + DN2 cells may be more activated than PLD4 − DN2 cells. Further studies are needed to decipher the difference between these two populations.

This study has some limitations. We did not analyze the transcriptional and clonal relationships between PLD4 + blasts and other B cell subsets; thus, it is unclear how PLD4 + blasts would be generated in the context of B cell development pathways or if PLD4 + blasts actually differentiate into ASCs in patients with SLE. Further studies including transcriptional may address such issues.

DN2 cells are likely a promising treatment target given its autoreactivity. However, the upregulated molecules in DN2 cells such as CD11c, CD19, and CXCR3 are also expressed in other immune cells. Therefore, specifically targeting DN2 cells without damaging other harmless cells is still challenging. As suggested in our experiments and the previous reports, the expression of *PLD4* is dominant in immune-related organs and is limited to pDCs and B cells among PBMCs. Although we mainly focused on B cells, pDCs are also regarded as a potential treatment target in SLE because they are primary sources of type I interferon [27, 28]. Blocking type I interferon has exhibited efficacy to ameliorate disease activity [29]. Furthermore, several clinical trials for monoclonal antibody drugs against pDCs are underway [30, 31]. Thus, we propose that PLD4 should be a novel cell surface marker for targeting both pDCs and pathological B cells, with potentially minimum side effects in SLE.

## Conclusions

This is the first research demonstrating that PLD4 + B cells are expanded in patients with SLE compared to HDs. The subpopulation of these cells overlapping with DN2 cells includes autoreactive ones and correlated with disease activity. In vitro study suggested that PLD4 + B cells are TLR-stimulated cells consistent with overlapping with DN2 cells. Thus, developing therapy targeting PLD4 should be a promising strategy in SLE.

### Supplementary Information

**Additional file 1.** cDNA sequence of human PLD4 proteins bound to Fc region of IgG2a.

**Additional file 2:** **Supplementary figure 1.** A. PLD4 mRNA expression levels were measured for multiple organ tissues’ cDNA (*n *= 1) by real time quantitative PCR. The bar graphs show relative expression levels of PLD4 normalized to GAPDH gene expression. Br: Brain, Col: Colon, Hrt: Heart, Kid: Kidney, Leu: Leukocyte, Liv: Liver, Mu: Muscle, Ov: Ovary, Pan: Pancreas, Pla: Placenta, Pro: Prostate, S.Int: Small intestine, Spl: Spleen, Tes: Testis, Thy: Thymus. B. The gating strategy of flow cytometry. Lymphocytes were defined using FSC-A and SSC-A followed by removal of doublets. pDCs were defined as cells negative for CD14, CD3, CD19, and CD11c and positive for BDCA-2. B cells were defined as CD19+ and CD3CD14CD16−. C. Representative histograms among the results of 3 HDs showing positivity of PLD4 in several subpopulations denoted above the panels.

**Additional file 3:** **Supplementary figure 2.** A. The gating strategy to define transitional B cells, naïve B cells, double negative B cells (DN), memory B cells, and plasmablasts. B. The boxplots show the ratios of each subpopulation in B cells are compared between HDs (*n* = 6) and SLE (*n* = 36). The Mann-Whitney U test was done. Tr = transitional B cells, NAV = naïve B cells, Mem = memory B cells, DN = double negative B cells, PB = plasmablasts. C. The boxplots show the ratios of each subpopulations accounting for either CD19+ B cells (red) or PLD4+ B cells (green). * = *P* < 0.05, *** = *P* < 0.005, by Mann−Whitney U test.

**Additional file 4:** **Supplementary figure 3.** A. In addition to double negative population, PLD4+ blasts included CD27+ memory and IgD naïve and transitional populations (middle). Both compartments were further analyzed for the ratios of CD11c+, CXCR5- cells (left and right). B. The scatter plots show the correlation between the ratios of PLD4+ blasts and the serum levels of serum C3 (left) or C4 (right) in SLE patients. The gray shade area represents the 95 % confidence interval of the regression line. Spearman rank correlation coefficient (ρ) and P values are shown.

**Additional file 5:** **Supplementary table 1.** The 10 largest heavy chain sequences from cultured PLD4+ blasts.

**Additional file 6:** **Supplementary table 2.** The 10 largest kappa chain sequences rom cultured PLD4+ blasts.

**Additional file 7:** **Supplementary table 3.** The 10 largest heavy chain sequences from cultured CD19+ B cells.

**Additional file 8:** **Supplementary table 4.** The 10 largest kappa chain sequences from cultured CD19+ B cells.

## Data Availability

The datasets used and/or analyzed during the current study are available from the corresponding author on reasonable request. On request, the corresponding author will provide the data that support the findings of this study. However, there are restrictions to the availability of anti-PLD4 monoclonal antibodies, as they are related to the material protected as a trade secret.

## Abbreviations

PLD4: Phospholipase D4
SLE: Systemic lupus erythematosus
pDC: Plasmacytoid dendritic cell
TLR: Toll-like receptor
BCR: B cell receptor
DN2: Double negative 2
