# Supplementary figures and images for: Phospholipase D4 as a signature of toll-like receptor 7 or 9 signaling is expressed on blastic T-bet + B cells in systemic lupus erythematosus
Source: Arthritis Res Ther. 2023 Oct 16;25:200. doi: 10.1186/s13075-023-03186-5 (PMC10577954; doi:10.1186/s13075-023-03186-5)

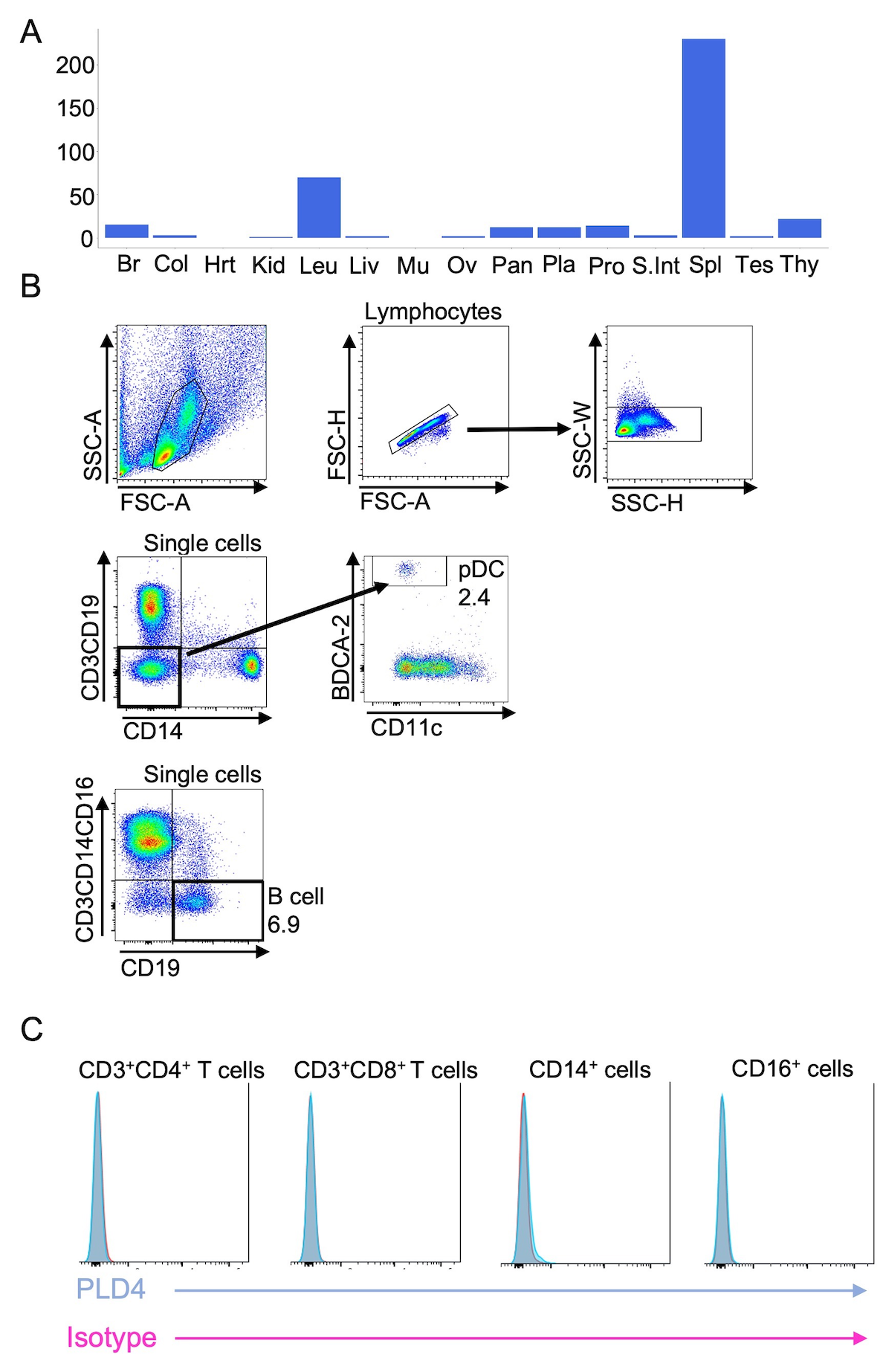

Supplement: Supplementary file 2 — Additional file 2: Supplementary figure 1. A. PLD4 mRNA expression levels were measured for multiple organ tissues’ cDNA (n = 1) by real time quantitative PCR. The bar graphs show relative expression levels of PLD4 normalized to GAPDH gene expression. Br: Brain, Col: Colon, Hrt: Heart, Kid: Kidney, Leu: Leukocyte, Liv: Liver, Mu: Muscle, Ov: Ovary, Pan: Pancreas, Pla: Placenta, Pro: Prostate, S.Int: Small intestine, Spl: Spleen, Tes: Testis, Thy: Thymus. B. The gating strategy of flow cytometry. Lymphocytes were defined using FSC-A and SSC-A followed by removal of doublets. pDCs were defined as cells negative for CD14, CD3, CD19, and CD11c and positive for BDCA-2. B cells were defined as CD19+ and CD3CD14CD16−. C. Representative histograms among the results of 3 HDs showing positivity of PLD4 in several subpopulations denoted above the panels. [file 13075_2023_3186_MOESM2_ESM.tiff]

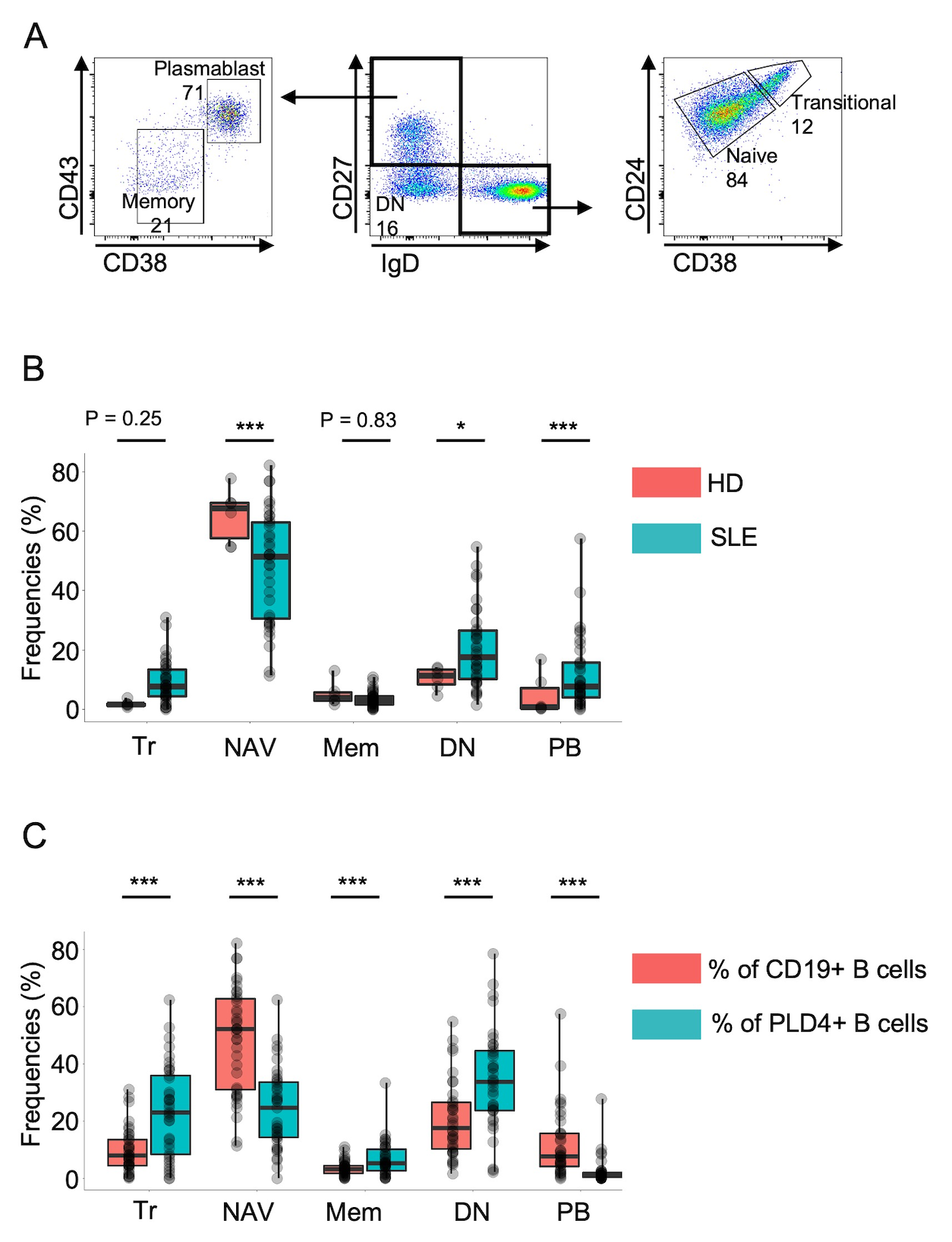

Supplement: Supplementary file 3 — Additional file 3: Supplementary figure 2. A. The gating strategy to define transitional B cells, naïve B cells, double negative B cells (DN), memory B cells, and plasmablasts. B. The boxplots show the ratios of each subpopulation in B cells are compared between HDs (n = 6) and SLE (n = 36). The Mann-Whitney U test was done. Tr = transitional B cells, NAV = naïve B cells, Mem = memory B cells, DN = double negative B cells, PB = plasmablasts. C. The boxplots show the ratios of each subpopulations accounting for either CD19+ B cells (red) or PLD4+ B cells (green). * = P < 0.05, *** = P < 0.005, by Mann−Whitney U test. [file 13075_2023_3186_MOESM3_ESM.tiff]

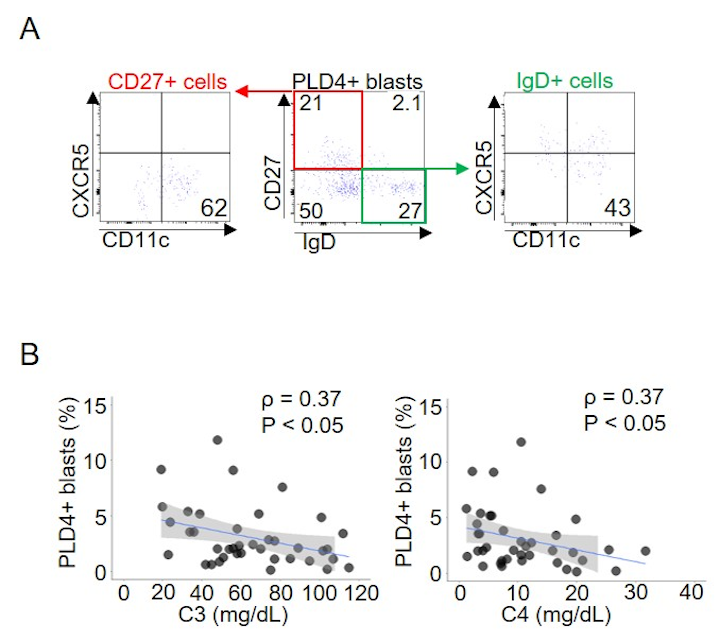

Supplement: Supplementary file 4 — Additional file 4: Supplementary figure 3. A. In addition to double negative population, PLD4+ blasts included CD27+ memory and IgD naïve and transitional populations (middle). Both compartments were further analyzed for the ratios of CD11c+, CXCR5- cells (left and right). B. The scatter plots show the correlation between the ratios of PLD4+ blasts and the serum levels of serum C3 (left) or C4 (right) in SLE patients. The gray shade area represents the 95 % confidence interval of the regression line. Spearman rank correlation coefficient (ρ) and P values are shown. [file 13075_2023_3186_MOESM4_ESM.tiff]
